# The role of TRPV1 ion channels in the suppression of gastric cancer development

**DOI:** 10.1186/s13046-020-01707-7

**Published:** 2020-10-02

**Authors:** Nannan Gao, Feng Yang, Siyuan Chen, Hanxing Wan, Xiaoyan Zhao, Hui Dong

**Affiliations:** 1 Department of Gastroenterology, Xinqiao Hospital, Third Military Medical University, Chongqing, 400037 China; 2 Department of Medicine, School of Medicine, University of California, San Diego, CA 92093 USA

**Keywords:** TRPV1 channel, Calcium signaling, Gastric cancer, Proliferation, Invasion, Metastasis

## Abstract

**Background:**

Although the aberrant expression and function of most Ca^2+^-permeable channels are known to promote gastrointestinal tumors, the association between transient receptor potential vanilloid receptor 1 (TRPV1) channels and gastric cancer (GC) has not yet been explored. Herein, we sought to determine the role of TRPV1 channels in the development of GC and to elucidate the underlying molecular mechanisms involved therein.

**Methods:**

Immunohistochemistry, qPCR, Western blot, immunofluorescence assays were used to detect the mRNA and protein expression of TRPV1 in GC cells and tissues, and the clinical significance of TRPV1 in GC was also studied by clinicopathologic analysis. CCK8, colony formation, flow cytometry assays were used to detect the proliferation and survival of GC cells, while transwell assay was used to detect migration and invasion of GC cells in vitro. Tumor xenograft and peritoneal dissemination assays in nude mice were used to examine the role of TRPV1 in GC development in vivo.

**Results:**

TRPV1 expression was significantly downregulated in human primary GC tissues compared to their adjacent tissues. The decreased expression of TRPV1 proteins in GC tissues was positively correlated with tumor size, histological grade, lymphatic metastasis, clinical stage, and was strongly correlated with poor prognosis of GC patients. Moreover, the expression of TRPV1 was closely correlated with Ki67, VEGFR, and E-cadherin, all of which are the well-known cancer markers for proliferation and metastasis. TRPV1 proteins were predominately expressed on the plasma membrane in several GC cell lines. TRPV1 overexpression blocked cell cycle at G1 phase to inhibit GC cell proliferation and attenuated migration and invasion of GC cells in vitro, but TRPV1 knockdown increased these parameters. TRPV1 significantly reduced gastric tumor size, number and peritoneal dissemination in vivo. Mechanistically, TRPV1 overexpression in GC cells increased [Ca^2+^]_i_, activated CaMKKβ and AMPK phosphorylation, and decreased expression of cyclin D1 and MMP2, while TRPV1 knockdown induced the opposite effects.

**Conclusions:**

TRPV1 uniquely suppresses GC development through a novel Ca^2+^/CaMKKβ/AMPK pathway and its downregulation is correlated with poor survival of human GC patients. Thus, TRPV1 upregulation and its downstream signaling may represent a promising target for GC prevention and therapy.

## Background

Gastric cancer (GC) is the second most common human cancer worldwide and is difficult to diagnose in its early stage [1]. GC is extremely difficult to cure once it metastasizes [2, 3]. Although the occurrence and progression of cancer are complex, numerous findings indicate that aberrant intracellular Ca^2+^ ([Ca^2+^]_i_) signaling is involved in the development of several types of gastrointestinal (GI) cancers, including GC and colon cancer [4]. Since plasma membrane Ca^2+^-permeable channels play important roles in the regulation of [Ca^2+^]_i_, their aberrant expression and function are positively associated with the occurrence and development of GI tumors [5, 6]. Consistently, we revealed that activation of G protein-coupled receptors (GPCRs), such as Ca^2+^ sensing receptors (CaSR) and vasoactive intestinal polypeptide (VIP) receptors, promotes GC progression via transient receptor potential vanilloid receptor 4 (TRPV4) channels and the Ca^2+^ signaling [7, 8]. Therefore, the Ca^2+^-permeable TRPV channels deserve further intensive investigation since they could be novel potential drug targets for GI tumor therapy [9].

The TRPV1 channel belongs to the Ca^2+^ permeable TRPV channel family and responds to noxious heat (> 43 °C), low pH value (< 5), capsaicin and so on [10–12]. The TRPV1 channel plays an important role in several physiological and pathological processes, such as nerve conduction, visceral pain sensing [9, 13, 14], and activation of immunity [15]. Furthermore, a few studies previously shown that TRPV1 was likely involved in tumor progression [16], and its activation reduced cell proliferation, migration and invasion in breast cancer [17], urothelial cancer [18] and papillary thyroid carcinoma [19]. However, little is currently known about the role of TRPV1 channel in GI tumorigenesis, except for Amaya G. et al., who reported that TRPV1 regulates neurogenic inflammation in the colon to presumably protect mice from colon cancer [20]. We also revealed that the TPRV1 channel inhibited EGFR-induced epithelial cell proliferation to prevent mice from developing colon polyps [21]. Although the expression of TRPV1 channel has been detected in rat gastric epithelial cells [22], almost nothing is known about its functional role in the upper GI epithelial cells, let alone its potential involvement in the pathogenesis of gastric disease. Importantly, the role of TRPV1 channel in gastric tumorigenesis has not been explored so far.

Aberrant [Ca^2+^]_i_ signaling contributes to multiple aspects of tumor progression such as cell proliferation, migration, invasion, apoptosis and autophagy [23, 24], and calmodulin (CaM) is one of the key proteins that triggers various signaling events in response to an increase in [Ca^2+^]_i_. Upon binding with Ca^2+^, CaM activates downstream calcium/calmodulin-dependent protein kinase kinases (CaMKK), including CaMKKα and CaMKKβ to further regulate adenosine mono phosphate activated protein kinase (AMPK). AMPK, a heterotrimeric Ser/Thr kinase, is well known to be involved in tumor progression [25]. Thr-172, as one of the important sites for AMPK activation, can be phosphorylated by CaMKKβ [25]. Several studies previously reported that AMPK inhibits proliferation and induces apoptosis in GC cells [26–28]. Although CaMKK has a well-established connection between Ca^2+^ signaling and cancer pathogenesis [29, 30], the role of aberrant Ca^2+^/CaMKKβ/AMPK signaling in GC progression and the underlying molecular mechanisms remain unexplored.

In the present study, we focused on the role of TRPV1 channels in GC progression and the underlying molecular mechanisms therein. Herein, we demonstrate for the first time that TRPV1 expression was decreased in primary human GC tissues, which was closely correlated with poor prognosis of GC patients. Moreover, TRPV1 could increase AMPK phosphorylation through Ca^2+^/CaMKKβ to downregulate the expression of cyclin D1 and matrix metalloproteinase-2 (MMP2), leading to the inhibition of GC cell proliferation, migration and invasion. Our results not only reveal the role of TRPV1 channel in GC suppression, but provide a novel insight to GC prevention and treatment.

## Materials and methods

### Ethics statement and human tissue samples

Twenty pairs of GC and adjacent tissues from the surgical patients in Xinqiao Hospital of Third Military Medical University (during 2016 and 2017) were used for real-time quantitative PCR, and all resected specimens were confirmed by pathological examination. Informed consent was obtained from all patients. All clinical studies were approved by the Clinical Research Ethics Committee of Third Military Medical University and were performed in accordance with approved guidelines. GC and adjacent tissue microarray for immunostaining were purchased from SHANGHAI OUTDO BIOTECH CO., LTD (Shanghai, China).

### Cell culture

MKN45, SGC7901, AGS, MGC803, BGC823 human gastric cancer cell lines and the human gastric normal epithelial mucosa cell line (GES-1) were purchased from Chinese Academy of Sciences (Shanghai, China). All cells were cultured in RPMI-1640 or DMEM-HIGH GLUCOSE medium (HyClone, USA) supplemented with 10% fetal bovine serum (HyClone, USA), 100 IU/mL penicillin and 100 μg/mL streptomycin (Invitrogen, USA). All cells were grown in a 37 °C humidified atmosphere containing 5% CO_2_.

### Preparation and infection of lentiviruses

Lentiviruses were purchased from Genechem Co., Ltd. (Shanghai, China). A lentivirus containing the full-length coding sequence (CDS) of TRPV1 (NM_080704) was designed to increase its expression in BGC823 cells, and lentiviral-based shRNA was used to silence the expression of TRPV1 in MKN45 cells. Sequences for TRPV1 shRNA and control were as follows: shRNA-1 (5′-GCATCTTCTACTTCAACTTCC-3′), shRNA-2 (5′-GGCCGACAACACGAAGTTTGT-3′) and control (5′-GTTCTCCGAACGTGTCACGT-3′). All shRNA groups that do not have a designed number used shRNA-1. Cells were infected with lentiviruses according to the protocol of the manufacturer. Briefly, cells were plated in 24-well plates at 1 × 10^5^ cells/well, lentiviruses were added into culture medium separately (the volume of lentiviruses was calculated as a MOI of 20), and medium were refreshed after 8 h. Puromycin was used to screen the stable cells after 72 h of lentivirus infection.

### Preparation and transfection of plasmids

The full length CDS of AMPK (NM_006251) was cloned into pcDNA3.1 to prepare overexpression plasmids; AMPK-siRNA sequence 5′-CTGCTTGATGCACACATGAAT-3′; CaMKKβ-siRNA sequence 5′- GTCAAGTTGGCCTACAATG-3′ were cloned into the GV102 vector separately to prepare siRNA-knocked down plasmids. Transfection of plasmids into cells was performed according to the protocol of FuGENE@ HD Transfection Reagent (Cat. No. E2311, Promega, USA). In brief, 1 × 10^5^ cells were cultured in each well of 24-well plates, and 1.5 μL of transfection reagent with 0.5 μg of plasmids were mixed and added to 500 μL culture medium. Cells were incubated with the mixture for 72 h.

### RNA extraction and real-time quantitative PCR (qPCR)

Total RNA was extracted from each group using the RNAiso Plus reagent (Cat. No. 9109, Takara, Japan). cDNA was synthesized using PrimeScript® RT-polymerase (Cat. No. R050A, Takara, Japan). Next, 50 ng of each cDNA was amplified as a template, and qPCR was performed using a SteponePlus device (Art. No. 272008342, Life Technologies, USA) with a SYBR® Premix Ex TaqTM II kit (Cat. No. RR820A, Takara, Japan). All samples were run in triplicate, and β-actin was used as an internal control. Data were quantified using the 2^−ΔΔCt^ relative quantitative method, normalized to β-actin expression, and expressed as the ratio of TRPV1 to β-actin mRNA levels. Primers were designed as follows:

TRPV1: 5′-TGGTATTCTCCCTGGCCTTG-3′ (forward)

5′- CTTCCCGTCTTCAATCAGCG-3′ (reverse)

AMPK: 5′-TGGTAGGAAAAATCCGCAGA-3′ (forward)

5′-CGACTTTCTTTTTCATCCAGC-3′ (reverse)

β-actin: 5′-GGCATCCACGAAACTACCTT-3′ (forward)

5′-CGGACTCGTCATACTCCTGCT-3′ (reverse)

### Immunohistochemistry (IHC)

Tissue samples were paraffin embedded and cut into 5 mm slices. After dewaxing and rehydration, tissue samples were incubated with anti-TRPV1 (Cat. No. ab3487, Abcam, UK) overnight at 4 °C after blocking. TRPV1 was detected using HRP-conjugated anti-rabbit secondary antibody (Cat. No. ZB-2301, ZSGB-BIO, China) and visualized with DAB. The negative control contained secondary antibody only. The gastric cancer tissue microarray we purchased also contains pathological score data for Ki67, VEGFR, E-cadherin and other proteins. The degree of staining in the TRPV1 sections was observed and scored by a pathologist. According to previously defined criteria [31, 32], the percentage of TRPV1 positivity was scored from 0 to 3 as follows: 0, < 10%; 1, 10–30%; 2, 30–50%; 3, > 50%. Staining intensity was scored according to a 4-point scale as follows: 0 (no staining); 1 (weak staining, light yellow); 2 (moderate staining, yellowish brown); and 3 (strong staining, brown). Subsequently, TRPV1 expression was calculated by multiplication of the percent positivity score and staining intensity score, resulting in a final score ranging from 0 to 9. Since TRPV1 was expressed as 0 in a large number of gastric cancer samples, expression of 0 was set as low expression and 1–9 as high expression. Ki67 was positively localized in the nucleus, VEGFR was positively localized in the cytoplasm and E-cadherin was positively localized in the plasma membrane. The scoring standard was the same as that described above.

### Immunofluorescence assay

Cells of each group were plated onto coverslips in 35 mm dishes and fixed in 4% polyformaldehyde for 15 min at room temperature. Coverslips were washed in PBS three times for 5 min. Cells were blocked in goat serum for 1 h at room temperature and then incubated with anti-TRPV1 antibody overnight at 4 °C. After three washes in PBS, cells were incubated with Cy3 labeled anti-rabbit (Cat. No. A0516, Beyotime, China) secondary antibody for 1 h at room temperature. Finally, nuclei were stained with DAPI for 10 min. Images were captured on a confocal microscope (Leica SP5, Germany).

### Western blot analysis

Whole-cell lysates were separated by SDS-PAGE on denaturing 10% or 12% gels and transferred to polyvinylidene fluoride membranes (Cat. No. ISEQ00010, Millipore, USA). Blots were blocked in 5% milk for 1 h at room temperature and then separately incubated at 4 °C overnight with the following specific primary antibodies: anti-TRPV1, anti-Ki67 (Cat. No. ab15580, Abcam, UK), anti-AMPK (Cat. No.5832, Cell Signaling Technology, USA), anti-phospho-AMPK (Cat. No.2535, Cell Signaling Technology, USA), anti-CaMKKβ (Cat. No. ab168818, Abcam, UK), anti-cyclin D1 (Cat. No. ab16663, Abcam, UK), anti-MMP2 (Cat. No.87809S, Cell Signaling Technology, USA), anti-β-catenin (Cat. No.8480, Cell Signaling Technology, USA), anti-phospho-β-catenin (Cat. No.9567, Cell Signaling Technology, USA), anti-AKT (Cat. No.9272, Cell Signaling Technology, USA), anti-phospho-AKT (Cat. No.4060, Cell Signaling Technology, USA), anti-ERK1/2 (Cat. No. ab184699, Abcam, UK), anti-phospho-ERK1/2 (Cat. No. ab214362, Abcam, UK), and anti-GAPDH (Cat. No. TA-08, ZSGB-BIO, China). All primary antibodies were diluted 1:1000. After rinsing, blots were incubated in HRP-conjugated anti-rabbit or anti-mouse (Cat. No. A0239 and A0216, Beyotime, China) secondary antibodies for 1 h at room temperature. Enhanced chemiluminescence (Cat. No. 34094, Thermo, USA) was used to detect immunoreactive bands. A human phospho-kinase array was used to detect changes in tumor-related signaling pathways (Cat. No. ARY003B, R&D Systems, USA), and 2 μM BAPTA-AM (Cas No. 126150–97-8, MedChemExpress, USA) was used to chelate intracellular calcium with cells being treated with BAPTA-AM for 2 h. Each experiment was performed in triplicate and repeated three times. The gray value of the bands was measured by ImageJ software for statistics.

### Cell proliferation assay

Cell viability and proliferation were measured by CCK8 assay (Cat. No.C0038, Beyotime Biotechnology, China). Cells were plated in 96-well plates (3000 cells/well) in triplicate and cultured for 0, 24, 48, 72 h or 96 h followed by addition of 100 μl of medium/CCK8 mixture (medium: CCK8, 9:1) 1–2 h before the endpoint of incubation. A Multiskan EX plate reader (Thermo Fisher Scientific, Germany) was used to quantify viable cells by measuring the absorbance at 450 nm, which estimates relative cell numbers rather than counting cells.

### Cell cycle analysis

Cells were digested, centrifuged and washed twice in cold PBS. The supernatant was subsequently discarded, and pre-cooled 70% ethanol was slowly added to the cell pellets that were kept at 4 °C overnight. After centrifugation the next day, the ethanol was removed, cells were washed once with PBS and were then incubated in a solution containing 0.2% Tween 20, 100 U/mL RNase, and 50 μg/mL propidium iodide for 20 min at 37 °C. Cell cycle analysis was performed using flow cytometry in which samples were gated on live cells with an excitation wavelength of 488 nm and an emission wavelength of 620 nm. LMD files were further analyzed using ModFit LT (Verity Software House, Topsham, ME).

### Colony formation assay

Long-term survival of cells was assessed by their ability to form colonies. Cells were plated in 6-well plates with 3 mL culture medium with 500 cells/well. After 10–12 days, the cell culture medium was removed, and cell clones were washed in PBS and fixed in 4% polyformaldehyde. Clone numbers were quantified after staining with crystal violet (Relative clonogenicity = Clone numbers/Average clone numbers of control group).

### Transwell migration and invasion assays

Twenty-four-well transwell chambers (Corning, USA) were used for this assay. 5 × 10^4^ cells were plated into each upper chamber with 8-μm pores and cultured in 200 μL serum-free RPMI-1640. The lower chambers were filled with 500 μL complete RPMI-1640 medium. After 24 h of incubation, cells that had migrated onto the lower surface were stained with crystal violet, and counted under a microscope (Olympus Corporation, Japan). The average value of three randomly selected fields was recorded as the number of migrated cells. Then, the upper surface of the polycarbonate filter was coated with 10% Matrigel (Collaborative Biomedical, USA), and 1 × 10^5^ cells were added to detect cell invasion. The other conditions were the same as those in the migration assay.

### Calcium measurement

Plating 1 × 10^4^ cells on coverslips, cells were loaded with 5 μM Fura-2 AM (Cat. No. F1221, Invitrogen, USA) in physiological salt solution (PSS) at 37 °C for 60 min and then washed in PSS or PSS with and 50 μM of the TRPV1 inhibitor SB-705498 (Cas No.501951–42-4, MCE, USA). Next, cells on coverslips were mounted in a standard perfusion chamber on a Nikon microscope stage. The ratio of Fura-2 fluorescence at 340 or 380 nm excitation (F340/380) was followed over time and captured with an intensified CCD camera (ICCD200) and a MetaFluor Imaging System (Universal Imaging, Downingtown, PA). PSS used in Ca^2+^ measurement contained the following: 140 mM Na^+^, 5 mM K^+^, 2 mM Ca^2+^, 147 mM Cl^−^, 10 mM HEPES, and 10 mM glucose at pH 7.4.

### Tumor xenograft and peritoneal dissemination assay in nude mice

The animal use protocol was approved by the Third Military Medical University Committee on Investigations Involving Animal Subjects. All animal care and experimental studies were conducted in accordance with the guidelines of the Animal Ethical Committee of Third Military Medical University and the Guide for the Care and Use of Laboratory Animals published by the US National Institutes of Health (NIH Publication No.8023, revised 1978). Animal studies were reported in compliance with the ARRIVE guidelines. First, 1 × 10^6^ TRPV1-overexpressing BGC823 cells and negative control (NC) cells were injected into the armpits of seven 4-week old male nude mice for tumor xenograft assay. TRPV1-overexpressing BGC823 cells were injected into the right armpit, and NC cells were injected into the left side. After 30 days of implantation, mice were sacrificed and tumor volumes in mm^3^ were calculated using the formula 1/2(length× width^2^). For the peritoneal dissemination assay, 1 × 10^6^ TRPV1-overexpression BGC823 cells and the NC cells were injected into the abdominal cavity of nude mice. Five weeks later, mice were sacrificed, and nodules were observed and quantified.

### Statistical analysis

SPSS Statistics 26.0 (IBM, USA) and Prism 8.0 (GraphPad, USA) software was used to analyze the data. All data are shown as MEANS ± SD. Pearson Chi-Square test was used to compare the correlations of TRPV1 expression and clinicopathological features. The relationship between TRPV1 expression and patient survival was examined by the log-rank test using the Kaplan-Meier method. Student’s t-test was used to analyze differences between two groups. One-way ANOVA was used to compare three or more groups. Significant differences are expressed in the figures and figure legends as *P < 0.05. All experiments were repeated using three biological replicates.

## Results

### Decreased TRPV1 expression in human primary GC tissues

**Fig. 1:**
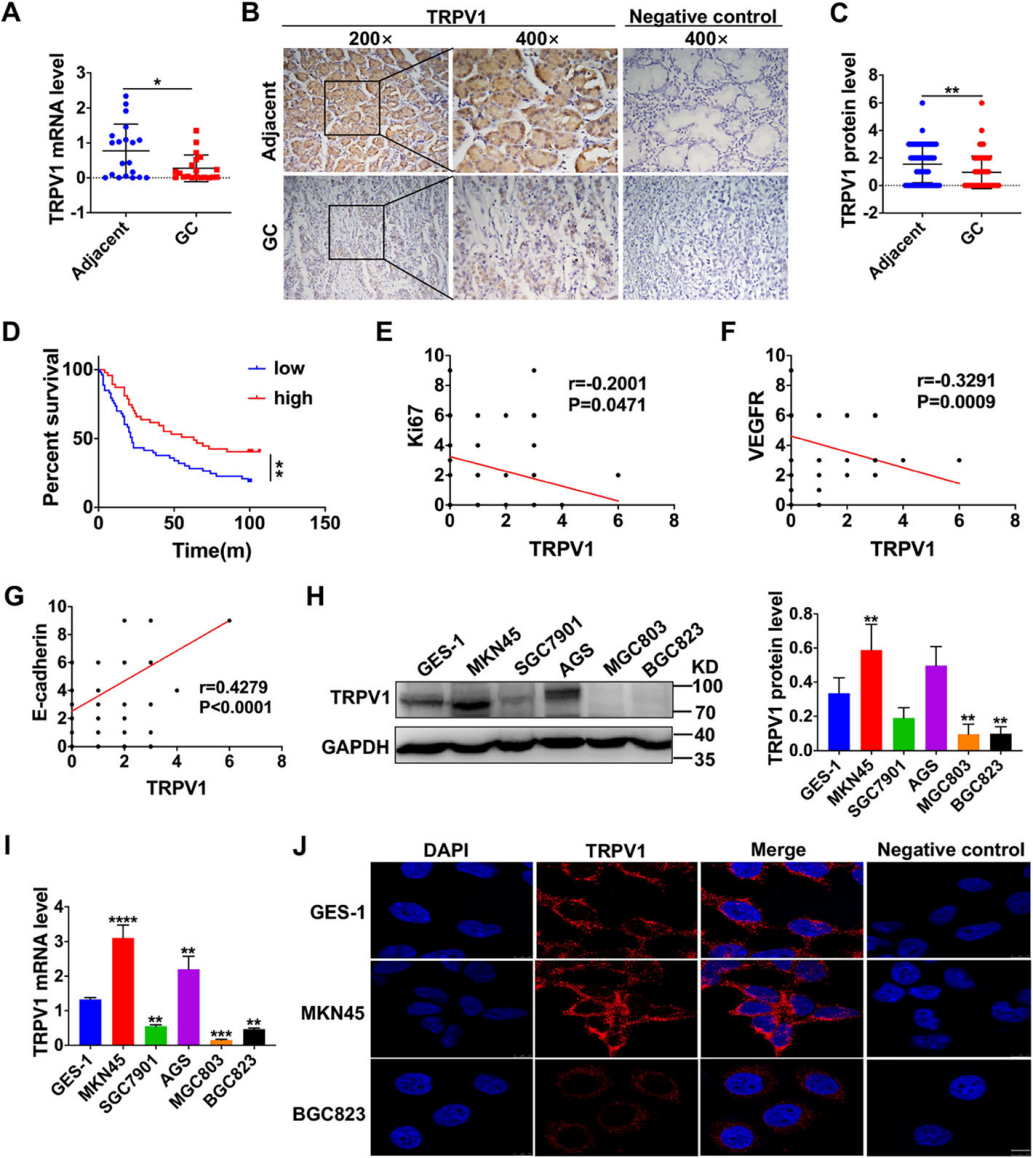
TRPV1 expression in gastric cancer and its correlation with clinical progression. **a** Transcript levels of TRPV1 detected by qPCR in human primary GC tissues and their adjacent tissues (*P < 0.05, *n* = 20 patients). **b** Representative images of immunohistological staining on TRPV1 proteins in GC tissues and adjacent tissues. **c** Summary data of immunohistological staining of TRPV1 proteins (***P < 0.001, *n* = 80 patients). **d** Analysis of survival rates in GC patients with low and high TRPV1 expression levels (**P < 0.01, *n* = 100 patients). Correlation between TRPV1 and Ki67 (**e**), VEGFR (**f**), and E-cadherin (**g**) expression (*P < 0.05, **P < 0.01, ***P < 0.001, ****P < 0.0001, *n* = 100 patients). **h** Western blot analysis of TRPV1 protein levels in a normal human gastric epithelial cell line (GES-1) and five GC cell lines (**P < 0.01 vs. GES-1, *n* = 3). **i** QPCR analysis of TRPV1 mRNA levels in GES-1 and nine GC cell lines (**P < 0.01, ***P < 0.001, ****P < 0.0001 vs. GES-1, *n* = 3). **j** Immunofluoresence staining images of TRPV1 proteins in GES-1, MKN45 and BGC823 cells. Negative control was not treated with primary antibody against TRPV1. Nuclei were stained in blue with DAPI. The white scale bars on the lower right are 7.5 μm. These images are representative of three independent experiments

The aberrant expression of ion channels is usually associated with the development of several human cancers. Since TRPV1 expression in human GC is lacking, we first predicted its expression from Oncomine tumor database, which indicated low expression of TRPV1 in GC (data not shown). Second, we collected human primary GC tissues and corresponding adjacent tissues to compare TRPV1 expression. As shown in Fig. 1a, TRPV1 expression was lower at transcriptional levels in human GC tissues than in adjacent tissues. Therefore, TRPV1 expression was decreased in human primary GC, consistently with the prediction from Oncomine tumor database.

Third, immunohistochemstry study was applied to detect protein expression of TRPV1 in human specimens from 100 patients with GC. Among these patients, their average age was 64 years old, 64% was male, 57% was diagnosed with advanced-stage (III/IV), and 73% had lymphatic metastasis (Table 1). As shown in Fig. 1b and c, the protein expression of TRPV1 was decreased in GC tissues compared to their adjacent tissues, but no staining was detected in the negative control without primary antibody, indicating its specific staining to TRPV1 proteins.

**Table 1:** Correlation between the TRPV1 expression and clinicopathological characteristics in 100 patients with GC

Characteristic	No. Patients (*n*=<100)	TRPV1 Expression		*Chi-square*	*P-value*
		Low No. (%)	High No. (%)		
Age					
<60 years	32	15 (46.9%)	17 (53.1%)	0.508	0.476
≥60 years	66	36 (54.5%)	30 (45.5%)		
Gender					
Male	64	31 (48.4%)	33 (51.6%)	1.486	0.223
Female	36	22 (61.1%)	14 (38.9%)		
Tumor size					
<5 cm	39	11 (28.2%)	28 (71.8%)	14.746	<0.001*
≥5 cm	59	40 (67.8%)	19 (32.2%)		
Histological grade					
G1/2	22	6 (27.3%)	16 (72.7%)	7.494	0.006*
G3/4	78	47 (60.3%)	31 (39.7%)		
Lymphatic metastasis					
Negative	27	7 (25.9%))	20 (74.1%)	10.884	0.001*
Positive	73	46 (63.0%)	27 (37.0%)		
Clinical stage					
I/II	42	11 (26.2%)	31 (73.8%)	20.288	<0.001*
III/IV	57	41 (71.9%)	16 (28.1%)		

To examine the involvement of TRPV1 in GC progression and development, the association between TRPV1 expression and clinicopathologic parameters of GC progression was subsequently analyzed. As shown in Table 1, the down-regulation of TRPV1 expression was correlated with the large tumor size (*P* < 0.001), high histological grade (*P* = 0.006), lymphatic metastasis (*P* = 0.001), and advanced clinical stage (*P* < 0.001), but not associated with patients’ age (*P* = 0.476), gender (*P* = 0.223). Furthermore, Kaplan-Meier analysis showed that the median survival times of GC patients with high and low TRPV1 expression were 63 and 22 months, respectively, indicating that the GC patients with high TRPV1 expression had a better prognosis, but those with low expression had a poor prognosis (*P* = 0.008, Fig. 1d). Therefore, the close association between TRPV1 expression and clinicopathologic parameters strongly suggests a role for TRPV1 in the progression and development of GC.

Since Ki67, VEGFR, and E-cadherin are well-recognized tumor markers for proliferation and metastasis of cancer cells, we further analyzed the association between these tumor markers and TRPV1 expression in GC. As shown in Fig. 1e–g, TRPV1 expression was negatively correlated with Ki67 (r = − 0.2001, *P* = 0.0471) and VEGFR (r = − 0.3291, *P* = 0.0009), but positively correlated with E-cadherin (r = 0.4279, *P* < 0.0001). Togetether, these data suggest that TRPV1 might be a tumor suppressor in human GC.

### TRPV1 expression in human GC cell lines

After analyzing the association between TRPV1 expression in human primary GC tissues and clinicopathologic parameters, we compared TRPV1 expression level among 5 GC cell lines (MKN45, SGC7901, AGS, MGC803 and BGC823) and a normal gastric mucosal cell line (GES-1). QPCR study showed that TRPV1 was expressed at varying degrees at the levels of transcripts in aforementioned all cell lines (Fig. 1i), which was further confirmed at the levels of proteins by immunoblotting analysis (Fig. 1h). The expression of TRPV1 was consistent at the levels of transcripts and proteins among these cell lines (Fig. 1h and i). Compared with normal GES-1 cells, MKN45 cells had the highest expression of TRPV1 but BGC823 cells had the lowest expression, indicating an obvious heterogeneity of GC cells [33].

We also performed immunofluorescence analysis to study the expression and localization of TRPV1 proteins. As shown in Fig. 1j, it was further confirmed for the highest TRPV1 expression in MKN45 cells but the lowest expression in BGC823 cells. Moreover, TRPV1 proteins were predominately expressed on the plasma membrane, but also in the cytoplasm at a small amount, consistently with the previous report that TRPV1 also existed in the endoplasmic reticulum [34]. Therefore, MKN45 and BGC823 cells were selected for further molecular and cell biological studies in the following experiments.

### Role of TRPV1 in inhibiting GC cell proliferation

**Fig. 2:**
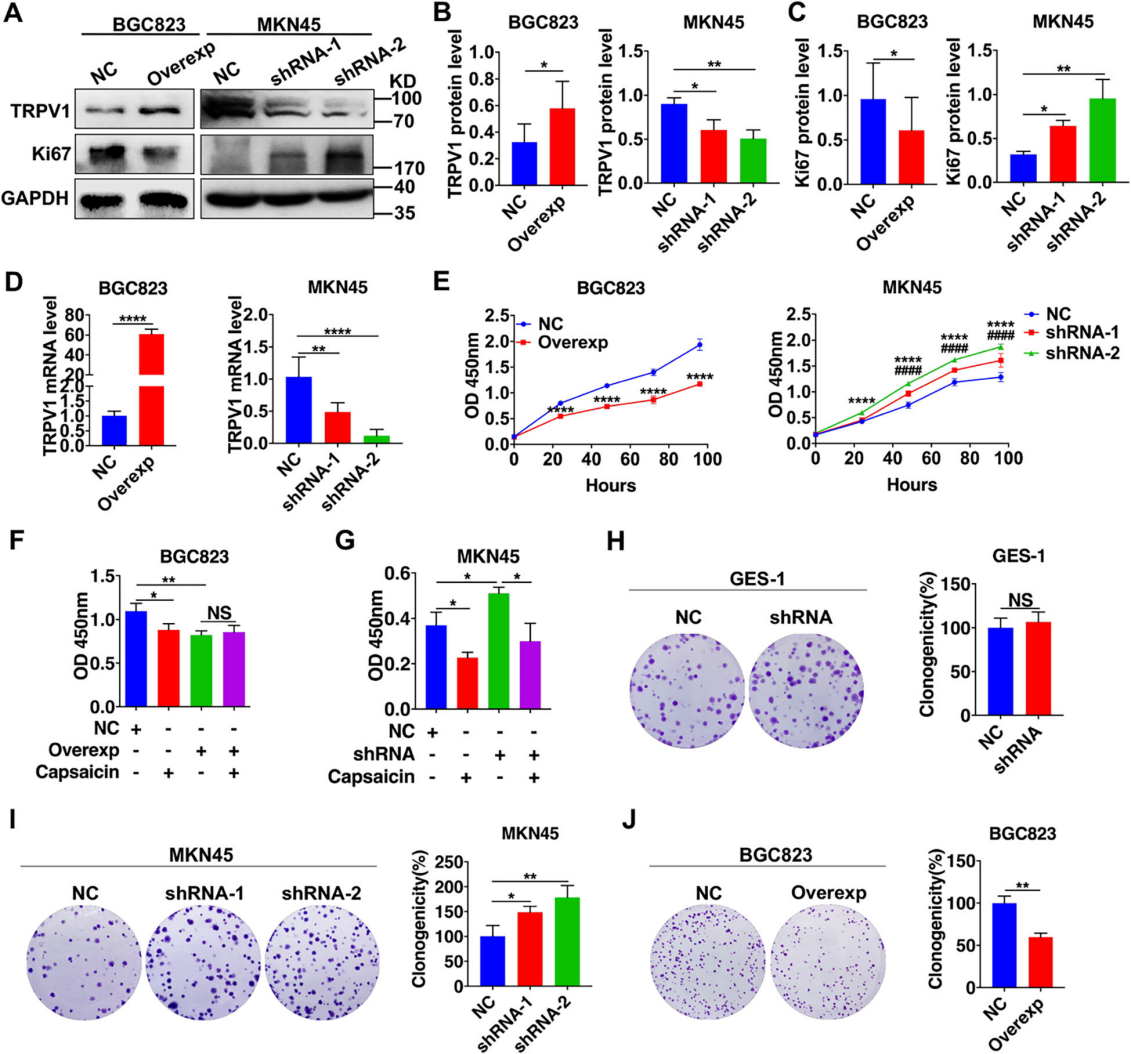
Effects of TRPV1 overexpression and knockdown on cell proliferation and clonogenicity. **a** Representative images of TRPV1 and Ki67 proteins in TRPV1-overexpressed BGC823 cells and TRPV1-knockeddown MKN45 cells. **b, c** The summary data of TRPV1 and Ki67 protein expression in GC cells (*P < 0.05, **P < 0.01, vs. NC, *n* = 3). **d** mRNA levels of TRPV1 detected by qPCR in TRPV1-overexpressed BGC823 cells and TRPV1-knockeddown MKN45 cells (**P < 0.01, ****P < 0.0001, vs. NC, *n* = 3). **e** GC cells proliferation in TRPV1-overexpressed BGC823 cells (left) and in TRPV1-knockeddown MKN45 cells (right) (shRNA-2 ****P < 0.0001, shRNA-1 ^####^*P* < 0.0001 vs. NC, *n* = 3). **f** Effects of TRPV1 overexpression or capsaicin (50 μM) alone and in combination on BGC823 cells proliferation (*P < 0.05, **P < 0.01, *n* = 3). NS: no significant difference. **g** Effects of TRPV1 knockdown or capsaicin (50 μM) alone and in combination on MKN45 cell proliferation (*P < 0.05, *n* = 3). **h, i** Effects of TRPV1 knockdown on clonogenicity of GES-1 and MKN45 cells (*P < 0.05, **P < 0.01 vs. NC, *n* = 3). **j** Effects of TRPV1 overexpression on clonogenicity of BGC823 cells (**P < 0.01 vs. NC, *n* = 3)

Our clinicopathologic study suggested that TRPV1 might be a human GC suppressor, and therefore its inhibition on GC cell proliferation was tested since high proliferation is a major characteristic of cancer cells. To this end, we applied lentiviruses infection to make TRPV1-overexpressed BGC823 cells and TRPV1-knockeddown MKN45 cells. As shown in Fig. 2a as representative image and Fig. 2b and c as summary data (*n* = 3 for each column), after successful overexpression or knockdown of TRPV1 in BGC823 or MKN45 cells at mRNA level (Fig. 2d) and protein level (Fig. 2a and b), respectively; proliferation marker Ki67 was decreased in TRPV1-overexpressed BGC823 cells but increased in TRPV1-knockeddown MKN45 cells compared with their corresponding negative controls (NC) (Fig. 2a and c). Therefore, our results obtained from GC cells are consistent with those obtained from primary GC tissues (Fig. 1e). Parallelly, TRPV1 overexpression decreased cell viability while TRPV1 knockdown increased it (Fig. 2e). Moreover, plate cloning experiments confirmed the inhibitory role of TRPV1 on clonogenicity in GC cells (Fig. 2i and j) but not in normal GES-1 cells with lower TRPV1 expression (Fig. 2h).

We further examined the effect of capsaicin (CAP), a well-known selective TRPV1 activator, on GC cell proliferation. As shown in Fig. 2f, although either CAP (50 μM) or TRPV1 overexpression alone inhibited GC cell proliferation with similar potency, a combination of them could not result in any superimposed inhibition. Moreover, CAP still inhibited proliferation in TRPV1-knockeddown MKN45 cells (Fig. 2g). Therefore, CAP suppression of GC cell proliferation was TRPV1-independent, as reported previously [35, 36]. Since capsaicin works on GC cells in a TRPV1-independent manner, we focused on the role of TRPV1 instead of CAP in the following experiments.

### TRPV1 suppression of GC growth by blocking cell cycle at G1 phase

**Fig. 3:**
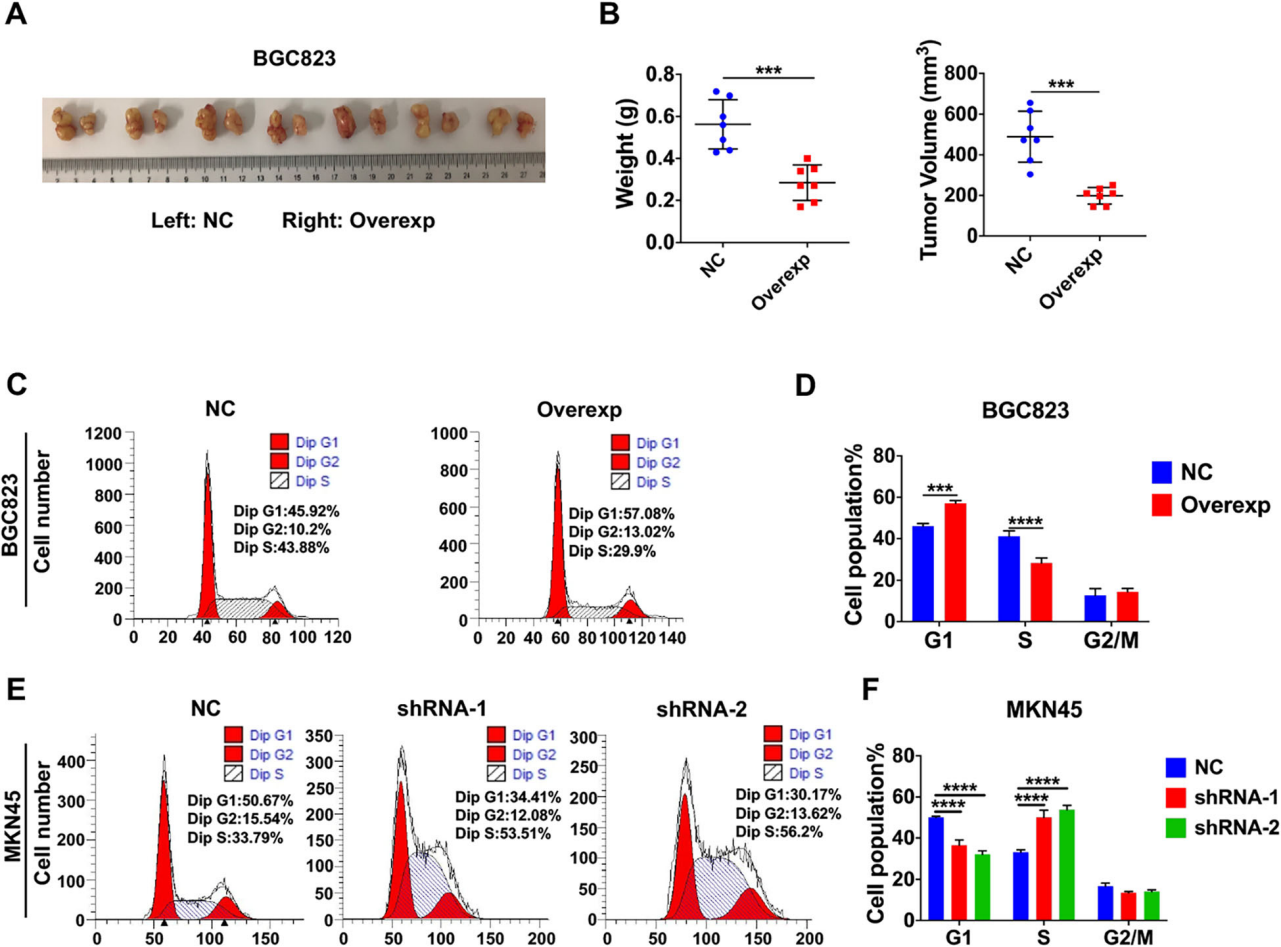
Effects of TRPV1 on GC growth in nude mice and GC cell cycle. **a** Images of subcutaneously xenografted gastric tumors in 7 nude mice. TRPV1-overexpressed BGC823 cells were implanted on the right side of mouse armpits, and NC cells were implanted on the left side. **b** Summary data of tumor weight (left) and volume (right) (***P < 0.001 vs. NC, *n* = 7 mice). Cell cycle analysis by flow cytometry in TRPV1-overexpressed BGC823 and NC cells (**c**), and summary data (**d**) (***P < 0.001, ****P<0.0001 vs. NC, *n* = 3). Cell cycle analysis by flow cytometry in TRPV1-knockeddown MKN45 and NC cells (**e**), and summary data (**f**) (****P < 0.0001 vs. NC, *n* = 3)

To further verify the role of TRPV1 in GC growth in vivo, xenografted GC model of nude mice was applied. In this model, the overexpression of TRPV1 in BGC823 cells markedly suppressed growth ability of GC cells after their implantation, leading to significant decreases in both tumor weights by about 52% and tumor volume by about 60% compared to their NC groups (Fig. 3a and b). Therefore, TRPV1 could not only inhibit GC cell proliferation in vitro but also suppress tumor growth in vivo, confirming our early notion that TRPV1 acts as a tumor suppressor in GC.

We further elucidated the underlying mechanisms of how TRPV1 inhibits GC cell proliferation in vitro and tumor growth in vivo. Since the cell cycle is an essential biological process that controls cell proliferation, we investigated how TRPV1 controls the GC cell cycle through genetic manipulation of TRPV1 expression and flow cytometry analysis [37]. As shown in Fig. 3c and d, compared to NC cells, the number of TRPV1-overexpressed BGC823 cells in G1 phase was increased by around 24%, and in S phase was decreased by around 31%. In contrast, the number of TRPV1-knockeddown MKN45 cells in G1 phase was decreased by around 30–40%, and in S phase was increased by around 50–60% (Fig. 3e and f). However, the G2 phase of the cell cycle were not significantly altered in response to manipulation of TRPV1 expression in GC cells. Therefore, mechanistically, TRPV1 blocked cell cycle at G1 phase to inhibit GC cell proliferation in vitro and tumor growth in vivo.

### TRPV1 suppression of GC cell migration and invasion both in vitro and in vivo

**Fig. 4:**
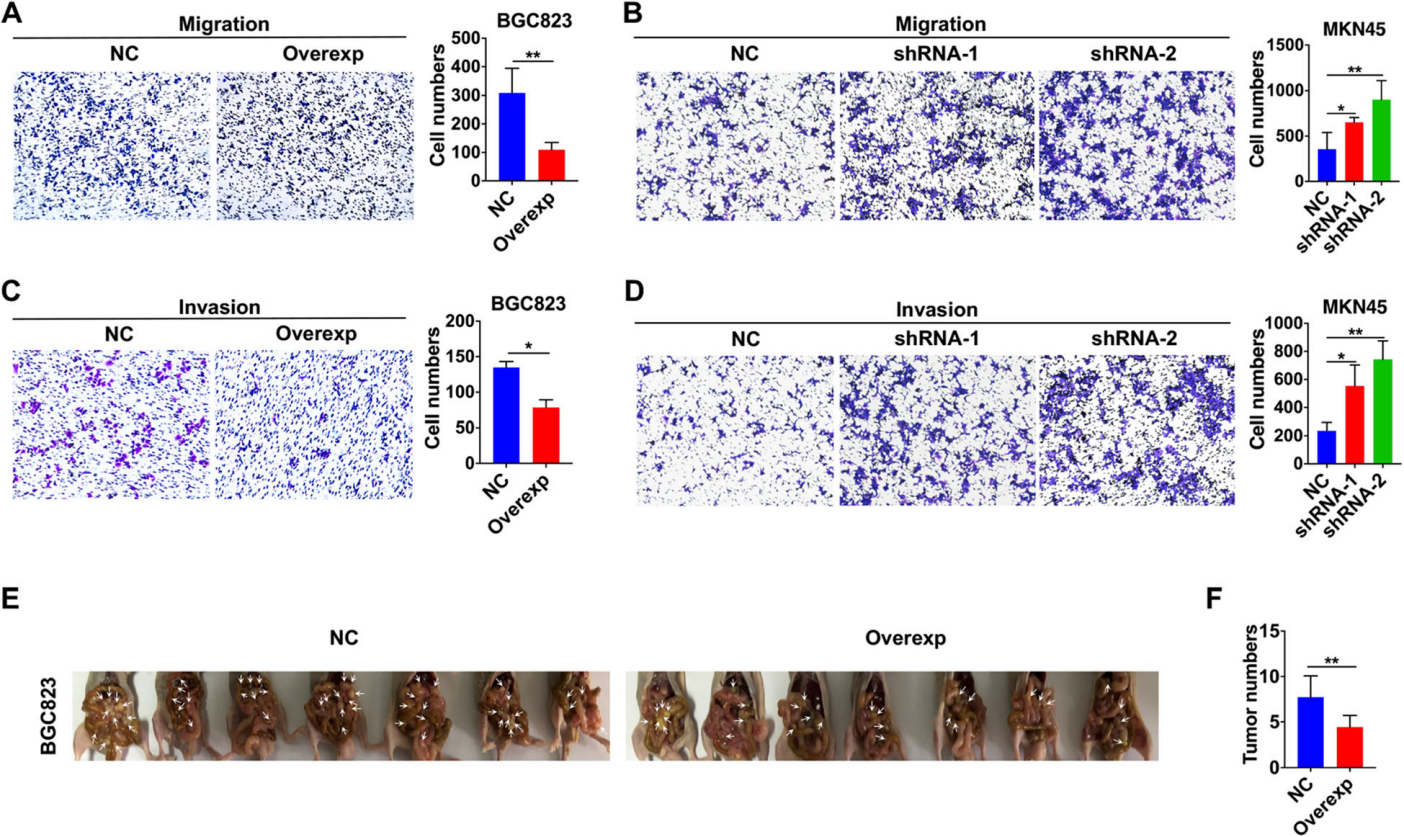
Effects of TRPV1 genetic manipulation on GC cell migration, invasion and metastasis. GC cell migration presented as original images and presented as summary data in TRPV1-overexpressed BGC823 cells (**a**) and TRPV1-knockeddown MKN45 cells (**b**) (*P < 0.05, **P < 0.01 vs. NC, *n* = 3). GC cell invasion presented as original images in and presented as summary data in TRPV1-overexpressed BGC823 cells (**c**) and TRPV1-knockeddown MKN45 cells (**d**) (*P < 0.05, **P < 0.01 vs. NC, *n* = 3). **e** Images of nude mice peritoneally injected with NC cells (left) and TRPV1-overexpressed BGC823 cells (right). **f** Summary data of tumor numbers from each groups of abdominal transplantation mouse model (**P < 0.01 vs. NC, *n* = 7 mice)

Since high migration and invasion of GC cells are not only their important characteristics but also major reasons to cause high mortality [38], we examined the role of TRPV1 in regulating GC cell migration and invasion. Our transwell assays showed that cell migration was reduced in TRPV1-overexpressed BGC823 cells, but enhanced in TRPV1-knockeddown MKN45 cells (Fig. 4a and b). Furthermore, changes in cell migration in both groups were over 50% compared to their NC groups (Fig. 4a and b). The trend of GC cell invasion was similar to that of migration (Fig. 4c and d), and the invasion in both groups was significantly altered at least 50% (Fig. 4c and d).

We applied abdominal transplantation tumor model of nude mice to verify the role of TRPV1 in suppressing GC invasion in vivo, and found that the overexpression of TRPV1 in BGC823 cells markedly suppressed GC cell metastasis after their peritoneal implantation (Fig. 4e), leading to a decrease in tumor numbers by about 40% compared to NC group (Fig. 4f). Therefore, TRPV1 suppressed GC cell migration and invasion both in vitro and in vivo.

### TRPV1-mediated signaling pathways in GC cells

**Fig. 5:**
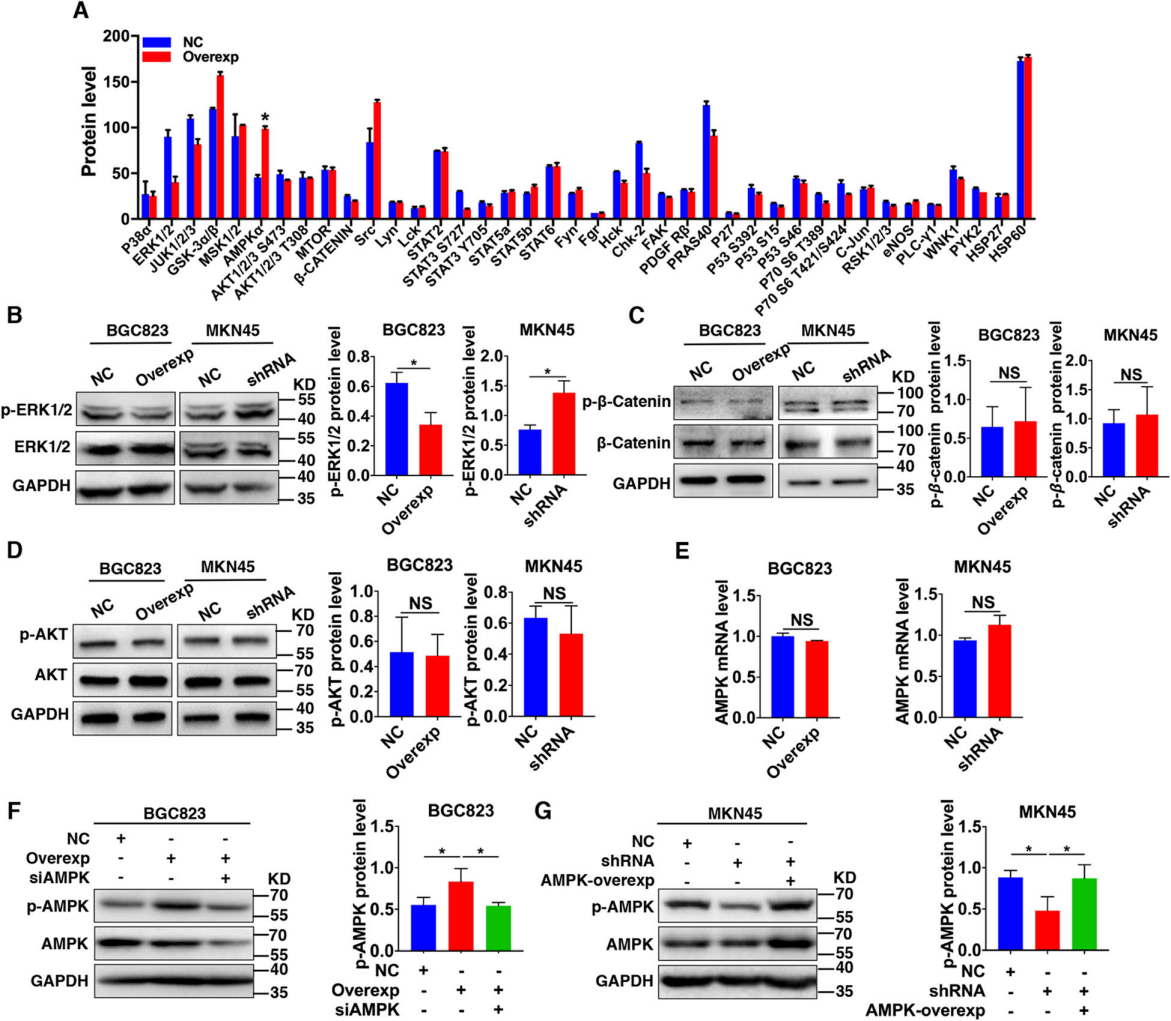
Changes in tumor-related signaling molecules after genetic manipulation of TRPV1 in GC cells. **a** Screening of phosphorylation of 40 key cancer-related signaling molecules analyzed by signaling pathway phosphorylation microarray after TRPV1 overexpression in BGC823 cells (*P < 0.05, *n* = 3). **b** ERK1/2 phosphorylation in TRPV1-overexpressed BGC823 cells and TRPV1-knockeddown MKN45 cells. Representative images are shown on the left and summary data on the right (*P < 0.05 vs. NC, *n* = 3). **c**, **d** Phosphorylation of β-catenin and AKT in TRPV1-overexpressed BGC823 cells and TRPV1-knockeddown MKN45 cells (NS: no significant difference, vs. NC, *n* = 3). **e** AMPK mRNA levels in TRPV1-overexpressed BGC823 or TRPV1-knockeddown MKN45 cells (NS: no significant difference, vs. NC, *n* = 3). **f** AMPK phosphorylation after TRPV1 overexpression in BGC823 cells and AMPK knockdown in TRPV1-overexpressed BGC823 cells (*P < 0.05, *n* = 3). **g** AMPK phosphorylation after TRPV1 knockdown in MKN45 cells and AMPK overexpression in TRPV1-knockeddown MKN45 cells (*P < 0.05, *n* = 3)

Since various cell signaling pathways are involved in GC tumorigenesis [39, 40], we initially screened TRPV1-mediated downstream signaling in GC cells. After screening the phosphorylation of 40 key proteins which are known to be closely related to tumorigenesis, we found that the phosphorylation of AMPK was significantly increased in TRPV1-overexpressed BGC823 cells compared to their corresponding negative controls (Fig. 5a), suggesting that AMPK was most likely involved. In addition, we assessed whether other key signaling proteins were also likely involved, including ERK1/2, β-catenin and AKT. As shown in Fig. 5b, TRPV1 overexpression attenuated but TRPV1 knockdown enhanced the phosphorylation of ERK1/2, suggesting TRPV1 suppresses ERK1/2 signaling in GC cells. However, due to the well-documented oncogenic role of ERK1/2 signaling in many cancers, including GC, we did not focus on it in further study. The other two signaling proteins, β-catenin and AKT were not significantly altered in response to genetic manipulation of TRPV1, excluding their involvements in TRPV1-mediated downstream signaling in GC cells (Fig. 5c and d).

### Critical role of AMPK phosphorylation in TRPV1-mediated signaling

We next focused on AMPK signaling since it is most likely involved in TRPV1-mediated downstream pathways in GC cells. This notion was further supported by investigation of AMPK phosphorylation. As shown in Fig. 5f and g, the phosphorylation levels at Thr-172 site of AMPK were markedly increased or decreased after TRPV1 was overexpressed in BGC823 cells or knocked down in MKN45 cells, respectively, compared to their corresponding NC; however, mRNA levels of AMPK were unchanged in both cell types (Fig. 5e). Therefore, TRPV1 likely stimulates AMPK phosphorylation in GC cells.

We further examined the critical role of AMPK in TRPV1 activation of GC cells after constructing AMPK overexpression and interference plasmids. As shown in Fig. 5f and g, compared to their corresponding NC, AMPK phosphorylation was markedly decreased by AMPK interference in TRPV1-overexpressed BGC823 cells, or increased by AMPK overexpression in TRPV1-knockeddown MKN45 cells. Therefore, TRPV1 indeed stimulates AMPK phosphorylation, which is critical in TRPV1-mediated signaling in GC cells.

### TRPV1 suppression of GC via AMPK activation but inhibition of Cyclin D1 and MMP2

**Fig. 6:**
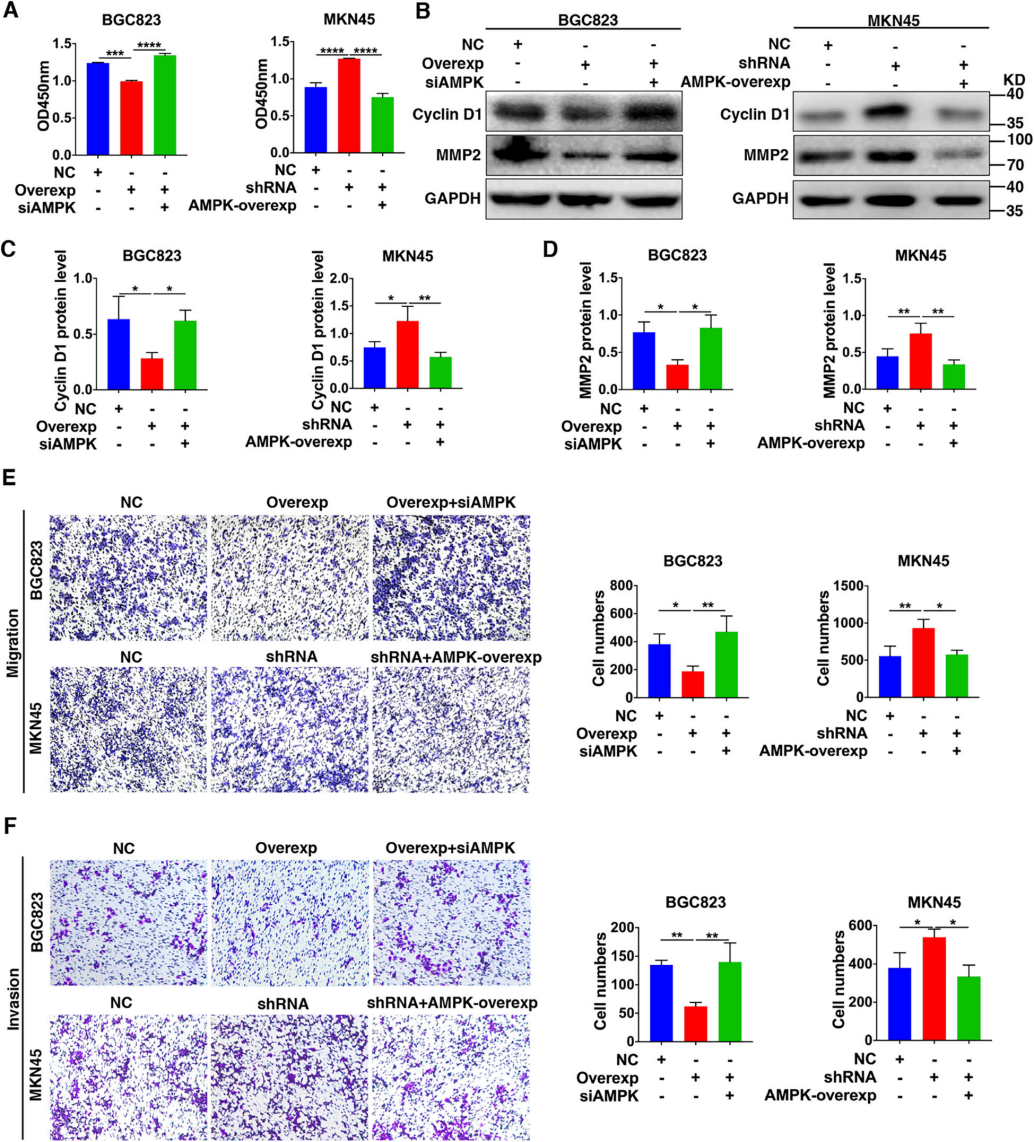
Effects of genetic manipulation of AMPK on GC cell proliferation, migration and invasion. **a** Effects of AMPK on cell proliferation in TRPV1-overexpressed BGC823 cells and in TRPV1-knockeddown MKN45 cells (***P < 0.001, ****P < 0.0001, *n* = 3). **b** Representative images of protein expression of cyclin D1 and MMP2 in TRPV1-overexpressed BGC823 cells transfected with AMPK-siRNA or TRPV1-knockeddown MKN45 cells transfected with AMPK-overexpression as well as their NC cells. **c, d** Summary data of cyclin D1 and MMP2 proteins in GC cells generated from the original data as in **b** (*P < 0.05, **P < 0.01, *n* = 3). **e**, **f** Effects of genetic manipulation of AMPK on cell migration and invasion in TRPV1-overexpressed BGC823 cells and TRPV1-knockeddown MKN45 cells. Representative images are shown on the left and summary data on the right (*P < 0.05, **P < 0.01, *n* = 3)

We further verified the role of AMPK in proliferation, migration and invasion of GC cells. As shown in Fig. 6a, reduced proliferation in TRPV1-overexpressed BGC823 cells could be recovered by siAMPK, but enhanced proliferation in TRPV1-knockeddown MKN45 cells could be reduced by AMPK overexpression. Similarly, reduced migration and invasion in TRPV1-overexpressed BGC823 cells were recovered by siAMPK, but the enhanced migration and invasion of TRPV1-knockeddown MKN45 cells were reduced by AMPK overexpression (Fig. 6e and f). Therefore, TRPV1 inhibited GC cell proliferation, migration and invasion through AMPK activation.

We elucidated the underlying mechanisms of how TRPV1-activated AMPK suppresses proliferation, migration and invasion in GC cells. Since both cyclin D1 and matrix metalloproteinase-2 (MMP2) play important roles in controlling the cell cycle and metastasis [41, 42], we investigated whether they are involved in TRPV1/AMPK-mediated suppression of GC cells. Western blotting analysis exhibited that compared to their corresponding NC, the expression of both cyclin D1 and MMP2 was decreased in TRPV1-overexpressed BGC823 cells, which were recovered by AMPK-siRNA (Fig. 6b–d). In contrast, the expression of both cyclin D1 and MMP2 was enhanced in TRPV1-knockeddown MKN45 cells, which was attenuated by AMPK overexpression (Fig. 6b–d). Therefore, TRPV1-activated AMPK suppressed GC development by inhibiting both cyclin D1 and MMP2.

### TRPV1 activation of AMPK phosphorylation via Ca^2+^/CaMKKβ pathway

**Fig. 7:**
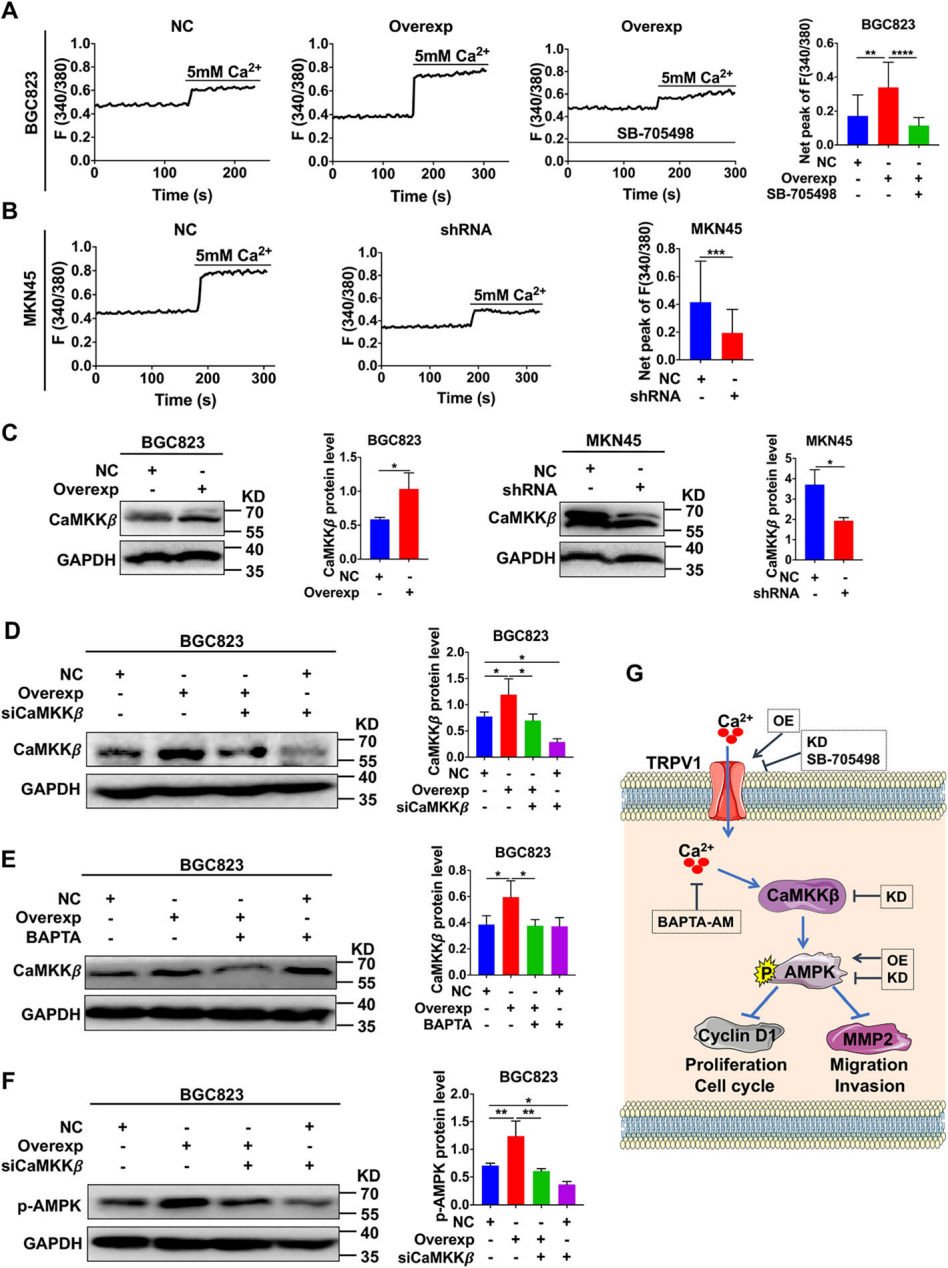
TRPV1/Ca^2+^-mediated GC suppression through activation of CaMKKβ/AMPK phosphorylation. **a** Representative time course of 5 mM CaCl_2_-induced [Ca^2+^]_i_ signaling in TRPV1-overexpressed BGC823 cells (middle) vs. NC (left) and TRPV1-overexpressed BGC823 cells treated with SB-705498 (50 μM) (right). Summary data are shown as a bar graph (**P < 0.01, ****P < 0.0001, *n* = 20 cells). **b** Representative time course of 5 mM CaCl_2_-induced [Ca^2+^]_i_ signaling in TRPV1-knockeddown MKN45 cells (middle) vs. NC (left). Summary data are shown as a bar graph (right) (***P < 0.001 vs. NC, *n* = 20 cells). **c** Expression levels of CaMKKβ proteins after TRPV1 overexpression in BGC823 cells or TRPV1 knockdown in MKN45 cells. Representative images are shown on the left and summary data on the right (*P < 0.05 vs. NC, *n* = 3). **d** Effects of CaMKKβ knockdown on CaMKKβ expression in TRPV1-overexpressed BGC823 or NC cells (*P < 0.05, *n* = 3). **e** Effects of BAPTA-AM (2 μM) on CaMKKβ expression in TRPV1-overexpressed BGC823 or NC cells (*P < 0.05, *n* = 3). **f** Effects of CaMKKβ knockdown on AMPK phosphorylation in TRPV1-overexpressed BGC823cells or NC cells (*P < 0.05, **P < 0.01, *n* = 3). **g** Proposed mechanisms of TRPV1-mediated GC suppression. The Ca^2+^ entry through TRPV1 channels causes CaMKKβ activation and AMPK phosphorylation that inhibits cyclin D1 and MMP2, leading to suppression of GC cell proliferation, migration and invasion. OE: overexpression, KD: knockdown, CaMKKβ: calcium/calmodulin-dependent protein kinase kinase β, AMPK: adenosine mono phosphate activated protein kinase, MMP2: matrix metalloproteinase-2

TRPV1 is a Ca^2+^–permeable channel [10] and CaMKKβ is a downstream kinase of calmodulin (CaM), a well-known intracellular Ca^2+^ binding protein [43]. Since CaMKKβ is an upstream AMPK activator [25], we hypothesized that AMPK activation requires the Ca^2+^/CaMKKβ in GC cells. To test this hypothesis, we first measured [Ca^2+^]_i_ in GC cells. Because CAP (50 μM) did not affect basal [Ca^2+^]_i_ in GC cells (data not shown), it is not a useful tool for the study on TRPV1 channel in these cells, as reported previously [35, 36]. Since TRPV channels are usually stimulated by GPCR activation, we stimulated Ca-sensing receptor (CaSR) with calcium (5 mM) for this purpose in GC cells [7]. Indeed, CaSR-mediated Ca^2+^ signaling was significantly increased in TRPV1-overexpressed BGC823 cells, which could be inhibited by SB-705498, a specific inhibitor of TRPV1 (Fig. 7a). In contrast, CaSR-mediated Ca^2+^ signaling was significantly decreased in TRPV1-knockeddown MKN45 cells (Fig. 7b). Thus, TRPV1 is a functional Ca^2+^–permeable channel to causes Ca^2+^ entry in GC cells.

We then examined the effect of TRPV1/Ca^2+^ on CaMKKβ, and found that the protein expression of CaMKKβ was markedly increased in TRPV1-overexpressed BGC823 cells, but decreased in TRPV1-knockeddown MKN45 cells compared with their NC (Fig. 7c). Furthermore, the increased CaMKKβ expression could be attenuated either by CaMKKβ-siRNA (Fig. 7d) or by pretreatment with [Ca^2+^]_i_ chelator, BAPTA-AM (2 μM) (Fig. 7e) in TRPV1-overexpressed BGC823 cells, in which the increased AMPK phosphorylation could be parallelly attenuated by CaMKKβ-siRNA (Fig. 7f). Therefore, TRPV1/Ca^2+^/CaMKKβ pathway is essential for AMPK phosphorylation in GC cells.

## Discussion

In the present study, we revealed that: (a) the expression of TRPV1 channel at the levels of mRNA and proteins was significantly decreased in human primary GC tissues; (b) the downregulation of TRPV1 expression was closely correlated with poor GC progression; (c) TRPV1 inhibited proliferation, migration and invasion of GC cells in vitro, and reduced gastric tumor size, number and peritoneal dissemination in vivo; and (d) mechanistically, TRPV1 suppresses GC progression through Ca^2+^/CaMKKβ/AMPK signaling pathway to reduce cyclin D1 and MMP2 expression.

We have verified that TRPV1 expression is downregulated in human primary GC tissues compared to their adjacent tissues, which is consistent with the prediction from the Oncomine tumor database. Importantly, we demonstrate for the first time that down-regulation of TRPV1 expression in GC tissues was correlated with large tumor size, high histological grade, lymphatic metastasis, advanced clinical stage and poor prognosis. Our clinicopathologic data strongly suggest a role for TRPV1 in the progression and development of GC. Moreover, TRPV1 expression in GC was positively correlated with E-cadherin expression, better prognosis and survival ratio of GC patients, but was negatively correlated with Ki67 and VEGFR expression. Since Ki67 is a well-known marker of cell proliferation, the faster tumor grows, the more sensitive to Ki67 [44]. While VEGFR promotes tumor angiogenesis [45], E-cadherin is closely related to tumor metastasis since the metastasis of tumor losses E-cadherin expression [46]. Therefore, TRPV1 could be a potential marker for GC prognosis due to its close association with GC progression.

We have provided further experimental data to support our notion that TRPV1 channel plays an important role in the progression and development of GC. The upregulation of TRPV1 expression attenuated GC cell proliferation, invasion and metastasis both in vitro and in vivo. In contrast, downregulation of TRPV1 expression promoted GC cell proliferation, invasion and metastasis. Therefore, TRPV1 channel seems to play a special role as GC suppressor because the aberrant expression and function of most Ca^2+^-permeable TRP channels are usually associated with GI tumor promotion [35, 47, 48]. It is well known that [Ca^2+^]_i_ is an important second messenger to regulate a wide range of cellular functions. The opening of TRP channels can promote Ca^2+^ entry which activates downstream signaling pathways, such as Ca^2+^/calmodulin kinase II (CaMKII), mitogen-activated protein kinase (MAPK), AMPK and so on to control cell proliferation, apoptosis and migration [49, 50]. Over the past two decades, several research groups including ours have identified six TRP channels (TRPC6, TRPM2, 5, 7, TRPV4, 6) that play an important role in GC development [7, 8, 47, 51–54]. However, all six of these TRP channels have been suggested as oncogene and tumor promoter in GC. Intriguingly, in contrast to the enhanced expression of these six TRP channels, TRPV1 channel expression is down-regulated in human GC and plays a suppressive role in GC. Therefore, it is important to elucidate the underlying molecular mechanisms for how TRPV1 as a Ca^2+^-permeable channel suppresses rather than promotes GC development.

Using phosphorylation chip screening for signaling pathway, we screened 40 key molecules that are closely related to cancer development. Interestingly, we found that phosphorylation level of AMPK was increased most significantly after overexpression of TRPV1 in GC cells, while ERK1/2 was decreased. Although TRPV1 regulation of AMPK is well studied in endothelial cells [55], smooth muscle cells [56], cardiomyocytes [57], and immune cells [58, 59], this has not been reported in digestive cancer cells. In the present study, we have provided sufficient evidence to demonstrate for the first time that TRPV1 is a Ca^2+^-permeable channel that uniquely suppresses GC development through activation of a novel CaMKKβ/AMPK pathway. The interesting role of TRPV1 channels in GC suppression needs further investigation, but it is not surprising since different Ca^2+^-permeable channels mediate cellular Ca^2+^ signals with various temporal and spatial precision, which may play different roles of anti-tumor or pro-tumor.

We revealed that cyclin D1 and MMP2 are the downstream molecules of AMPK activation to finally inhibit both proliferation and migration of GC cells. Cyclin D1 has been recognized as a proto-oncogene to promote cell cycle from G1 phase to S phase by activating CDK4, a cyclin-dependent kinase specific to G1 phase. Some studies have reported that AMPK inhibits GC growth via cyclin D1 suppression [41, 60]. As is known, the metastasis is a crucial characteristic for the late stages of cancer [42, 61]. MMP2 belongs to the zinc-dependent metalloproteinase gene family and plays a critical role in cancer metastasis [62]. AMPK, an upstream regulator of MMP2 [63], could decrease the migration and invasion of colorectal cancer through inhibition of MMP2 [64].

Capsaicin has been recognized as an anti-cancer agent in variety of cancers due to its apoptotic effect and inhibitory effect on cancer cell growth, metastasis and tumor angiogenesis [65]. Although capsaicin is a commonly used TRPV1 agonist in neurons used to induce Ca^2+^ influx, its mechanism of action in tumorigenesis is complex. Capsaicin directly activates PI3K/AKT and PKA in a TRPV1/Ca^2+^-independent manner [66, 67]. Moreover, capsaicin activates TRPV6 instead of TRPV1 to induce apoptosis in GC and lung cancer cells [35, 36]. In the present study, we found that capsaicin could not induce Ca^2+^ signaling in GC cells expressing functional TRPV1 channels at high concentration of 50 uM (comparing the IC_50_ of 0.5 uM in many other cell types) [36]. One possibility is loss of TRPV1 channel function in GC cells. However, this is not the case since: 1) pharmacological blocker and genetic manipulation of TRPV1 could alter [Ca^2+^]_i_ in GC cells, 2) genetic manipulation of TRPV1 could alter GC cell proliferation, migration and invasion both in vitro and in vivo, and 3) [Ca^2+^]_i_ chelator BAPTA-AM efficiently prevented TRPV1-mediated CaMKKβ activation and AMPK phosphorylation in GC cells. Another possibility is an aberrant functional alteration/mutation of TRPV1 channel in GC cells that causes a loss of TRPV1 sensitivity to capsaicin, which needs further investigation. Due to the fact that TRPV1 can be stimulated by a variety of substances [10, 11], TRPV1 channels in GC cells could still be activated by the other non-capsaicin agents, such as gastric acid and heating diets.

## Conclusions

We demonstrate for the first time that TRPV1 channel uniquely suppresses GC development in vitro and in vivo. Mechanistically, TRPV1/Ca^2+^ activates CaMKKβ/AMPK pathway to consequently decrease the expression of cyclin D1 and MMP2 (Fig. 7g). Moreover, TRPV1 channel loses its sensitivity to capsaicin, suggesting a possible functional alteration/mutation of TRPV1 channel in GC cells. Although TRP channels represent a relatively new field of cancer research with most studies still in their infancy, these channels hold tremendous potential that has yet to be uncovered in the hopes of achieving major clinical breakthroughs in GC therapy. Particularly, the decreased expression of TRPV1 has potentially diagnostic and prognostic significance for human GC, and upregulation/recovery of TRPV1 expression, function and its downstream signaling may be a novel promising strategy for prevention/therapy of GC.

## Data Availability

Not applicable.

## Abbreviations

TRPV1: Transient receptor potential vanilloid receptor 1
CaMKKβ: Calcium/calmodulin-dependent protein kinase kinase β
AMPK: Adenosine mono phosphate activated protein kinase
MMP2: Matrix metalloproteinase-2
OE: Overexpression
KD: Knockdown
shRNA: Short hairpin RNA
